# Testing avian compass calibration: comparative experiments with diurnal and nocturnal passerine migrants in South Sweden

**DOI:** 10.1242/bio.20149837

**Published:** 2014-12-12

**Authors:** Susanne Åkesson, Catharina Odin, Ramón Hegedüs, Mihaela Ilieva, Christoffer Sjöholm, Alexandra Farkas, Gábor Horváth

**Affiliations:** 1 Department of Biology, Centre for Animal Movement Research, Lund University, Ecology Building, SE-223 62 Lund, Sweden; 2 Max Planck Institute for Informatics, Campus E1.4, D-66123 Saarbrücken, Germany; 3 Institute of Biodiversity and Ecosystem Research, Bulgarian Academy of Sciences, 1113 Sofia, Bulgaria; 4 Environmental Optics Laboratory, Department of Biological Physics, Physical Institute, Eötvös University, H-1117 Budapest, Pázmány sétány 1, Hungary; 5 Manao Group, INRIA Sud-Ouest Bordeaux, 33400 Talence Cedex, France

**Keywords:** orientation, migratory birds, magnetic compass, compass calibration, passerines, *Acrocephalus schoenobaenus*, *Prunella modularis*, *Erithacus rubecula*

## Abstract

Cue-conflict experiments were performed to study the compass calibration of one predominantly diurnal migrant, the dunnock (*Prunella modularis*), and two species of nocturnal passerine migrants, the sedge warbler (*Acrocephalus schoenobaenus*), and the European robin (*Erithacus rubecula*) during autumn migration in South Sweden. The birds' orientation was recorded in circular cages under natural clear and simulated overcast skies in the local geomagnetic field, and thereafter the birds were exposed to a cue-conflict situation where the horizontal component of the magnetic field (mN) was shifted +90° or −90° at two occasions, one session starting shortly after sunrise and the other ca. 90 min before sunset and lasting for 60 min. The patterns of the degree and angle of skylight polarization were measured by full-sky imaging polarimetry during the cue-conflict exposures and orientation tests. All species showed orientation both under clear and overcast skies that correlated with the expected migratory orientation towards southwest to south. For the European robin the orientation under clear skies was significantly different from that recorded under overcast skies, showing a tendency that the orientation under clear skies was influenced by the position of the Sun at sunset resulting in more westerly orientation. This sun attraction was not observed for the sedge warbler and the dunnock, both orientating south. All species showed similar orientation after the cue-conflict as compared to the preferred orientation recorded before the cue-conflict, with the clearest results in the European robin and thus, the results did not support recalibration of the celestial nor the magnetic compasses as a result of the cue-conflict exposure.

## INTRODUCTION

Migratory songbirds are able to use geomagnetic information and celestial cues involving the position of the Sun and stars as well as the pattern of skylight polarization for orientation (e.g. Emlen, 1975; Able, 1980; Wiltschko and Wiltschko, 1995). In addition, birds possess an inherited magnetic compass based on the angle of inclination and not the polarity of the Earth's magnetic field (Wiltschko and Wiltschko, 1972), encoding the population-specific migratory direction and interacting with the rotation centre of the stellar sky (Weindler et al., 1996). In some areas, especially near the Polar Regions, migratory birds face a conflict between the directions given by the Sun and stellar compass compared to the magnetic compass, since geomagnetic declination exhibits large variations between nearby sites (Skiles, 1985). To cope with these difficulties, birds have been proposed to recalibrate their compasses both during ontogeny and adult life (Able and Able, 1990a; Able and Able, 1990b; Able and Able, 1993; Able and Able, 1995; Muheim et al., 2003). There is also the possibility that birds show local adaptations in their orientation system affecting the functional characteristics of their compass systems, and how they perform these compass calibrations (Åkesson, 1994; Åkesson et al., 2002).

Recently, Muheim et al. showed that migratory Savannah sparrows, *Passerculus sandwichensis* use the lowest part of the sky to recalibrate their magnetic compass (Muheim et al., 2006a; Muheim et al., 2008), while other studies by Wiltschko et al., Gaggini et al., Chernetsov et al. and Schmaljohann et al. have been unable to find the same response in other species of passerines tested in other geographical regions (i.e. Tasmanian Silvereyes, *Zosterops l. lateralis* in Australia, European robins, *Erithacus rubecula* in Germany, Pied flycatchers, *Ficedula hypoleuca* in Italy, Song thrushes, *Turdus philomelos* in Russia, Northern wheatears, *Oenanthe oenanthe* in Germany) (Wiltschko et al., 2008; Gaggini et al., 2010; Chernetsov et al., 2011; Schmaljohann et al., 2013). Also the white-throated sparrows, *Zonotrichia albicollis*, migrating across North America, have been demonstrated to recalibrate the magnetic compass on the basis of polarized skylight near the horizon (Muheim et al., 2009). Prior to the Muheim et al. study (Muheim et al., 2006a), Sandberg et al. performed cue-conflict experiments with a number of North American passerines in Alabama, resulting in interesting differences in orientation between cage and release experiments, but also similarities between species in how the investigated species recalibrated their celestial compass based on geomagnetic information (Sandberg et al., 2000). Åkesson et al. found similar results from cue-conflict exposures with White-crowned sparrows, *Zonotrichia leucophrys gambelli* in Northern Canada, where the birds followed the magnetic shift closely, but did select a recalibrated course afterwards at sunset in the local geomagnetic field (Åkesson et al., 2002). However, experiments after cue-conflict were only performed in the local geomagnetic field, thus it was impossible to say which compass they had recalibrated (Åkesson et al., 2002). Results from cue-conflict experiments with caged birds have resulted in different outcomes and have repeatedly been reviewed by several authors discussing alternative explanations of the so far incoherent results (Able, 1993; Åkesson, 1994; Wiltschko et al., 1998; Wiltschko and Wiltschko, 1999; Muheim et al., 2006b).

Taken together, there are substantial variations in the responses to cue-conflict exposures, between species, geographical areas and experiments. It is possible that some of these variations may be explained by the different methods used (Helbig, 1990; Åkesson, 1994; Åkesson, 2003). Furthermore, strictly comparative orientation tests have so far only been performed to a limited degree reporting mainly similar responses between species when tested at the same site (e.g. Åkesson, 1994; Åkesson and Bäckman, 1999), but also different responses in the same species between sites (Sandberg et al., 1988; Sandberg et al., 1991; Åkesson, 1993; Åkesson et al., 1995; Åkesson et al., 2001; Åkesson et al., 2005). Hence, there is an urgent need for carefully designed comparative experiments with various species of birds to investigate if possibly migration ecology may at least in part be an explanation of the different outcomes.

In order to investigate whether there might be differences in compass calibration responses between species captured and tested at the same location in South Sweden, we have performed comparative orientation cage experiments with three species of passerine migrants, two of which are nocturnal migrants (European robin, *Erithacus rubecula* and sedge warbler, *Acrocephalus schoenobaenus*) and one which predominantly migrates at daytime (dunnock, *Prunella modularis*). The experiments were performed outdoors with the same cages and test procedure for registering orientation for each species. Tests were done at sunrise for dunnocks and at sunset for European robins and sedge warblers. The birds' orientation was recorded under natural clear and simulated overcast skies and thereafter they were exposed to a cue-conflict situation (a shifted magnetic field mN +90° or −90°) at the following sunrise and sunset. After the initial tests and cue-conflict the orientation for each bird was recorded under clear skies without meaningful magnetic information in a vertical magnetic field, and in the local geomagnetic field without any visual compass cues available under simulated overcast skies. All orientation experiments were performed at the same time relative to sunrise and sunset for each species, respectively. At the cue-conflict exposures and during the experiments under clear skies we measured the patterns of skylight polarization by full-sky imaging polarimetry in order to characterize the availability of polarized skylight to the birds at exposure. We set up a hypothesis that the birds would re-calibrate their magnetic compass on the basis of polarized skylight, and according to the null hypothesis we expected no difference in orientation before and after the cue-conflict in relation to geomagnetic north.

## RESULTS

We found high migratory activity for our experimental birds, i.e. 89.4% of the experiments resulted in registrations of migratory activity (>40 registrations) with mean orientation significantly different from random in the orientation funnels (Table 1). Only 43 (8.9%) of the tests were classified as non-active and in 8 (1.7%) of the tests the birds were disoriented showing no meaningful orientation in the cages. In 84.3% of all tests the sum of both indexes was ≧3. Out of a total of 483 experiments 56 (11.6%) of the tests resulted in an axial distribution with r_2_>r. There was no significant difference in orientation of the dunnocks tested in 2008 and 2009 under natural clear skies (Watsons's U^2^ test: U^2^ = 0.06, p>0.5) and in simulated overcast conditions (Watsons's U^2^ test: U^2^ = 0.04, p>0.5), and therefore the two groups were pooled in further analyses.

**Table 1.**
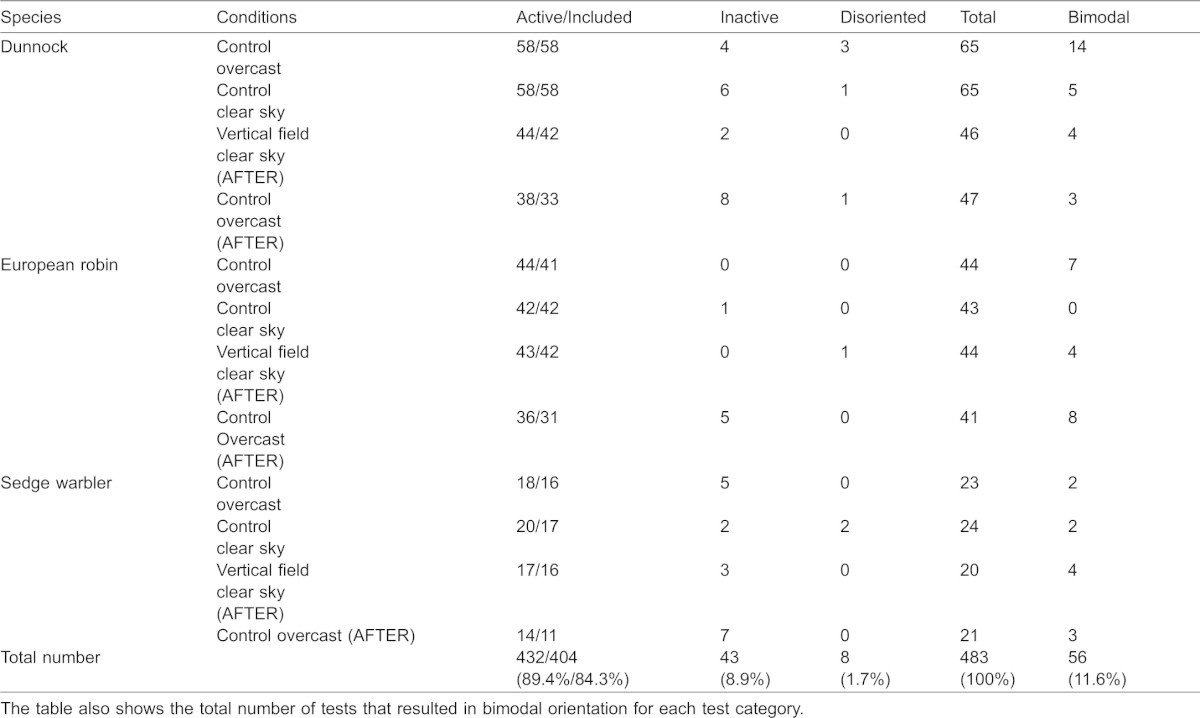
Number of active, inactive and orientation tests classified as disoriented for each experimental category and species (dunnock, European robin, sedge warbler) in autumn

### Orientation under natural clear skies

**Fig. 1.**
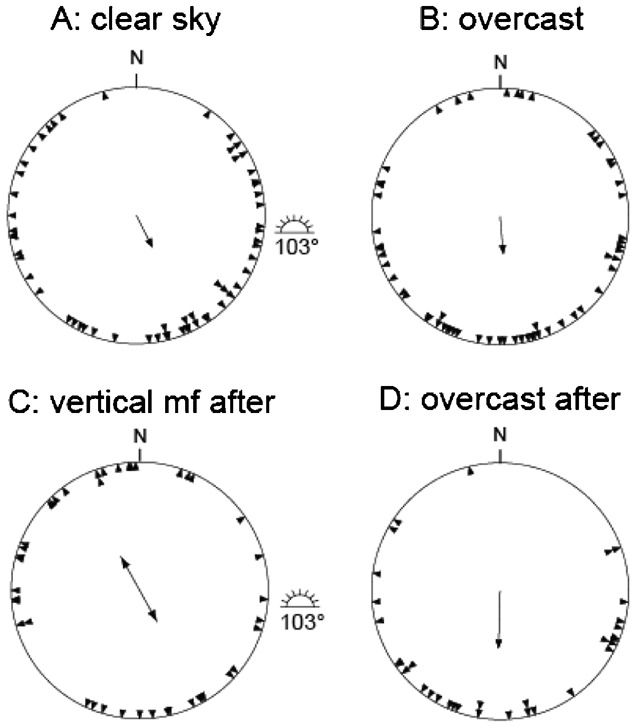
Results from our outdoor orientation cage experiments with juvenile dunnocks, *Prunella modularis*, at Stensoffa Ecological Field Station in South Sweden during autumn migration. Given is the mean angle of orientation (α) as indicated by a vector for different experimental conditions: (A) clear sky, (B) overcast sky, (C) exposure to clear skies without magnetic cues at vertical magnetic field vector, and (D) simulated overcast as a control after cue-conflict exposure. The azimuth to the Sun (¤) in the middle of the test hour is given. The mean orientation for each individual bird is shown as a triangle within the circle. Circular statistics for each circular distribution is given in Table 2.

**Fig. 2.**
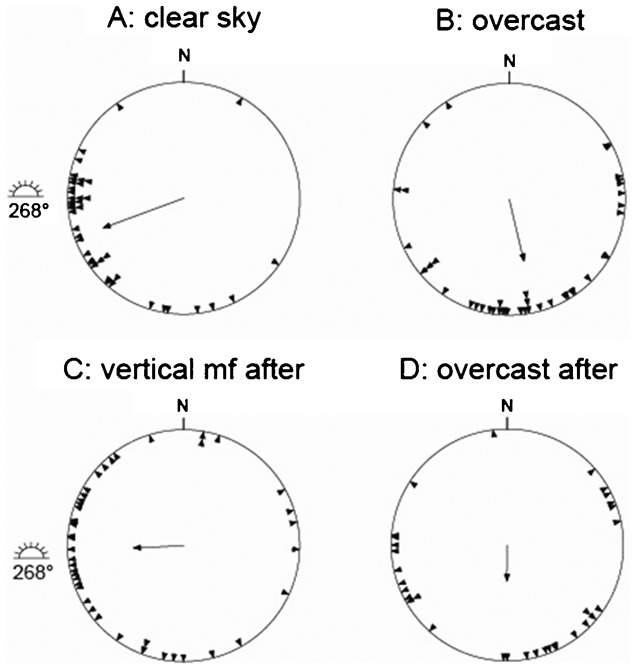
Results from our outdoor orientation cage experiments with juvenile European robins, *Erithacus rubecula* at Stensoffa Ecological Field Station in South Sweden during autumn migration. (A) Clear sky, (B) overcast sky, (C) exposure under clear skies without magnetic cues at vertical magnetic field vector, and (D) simulated overcast as a control after cue-conflict exposure. For further information, see legend of Fig. 1.

**Fig. 3.**
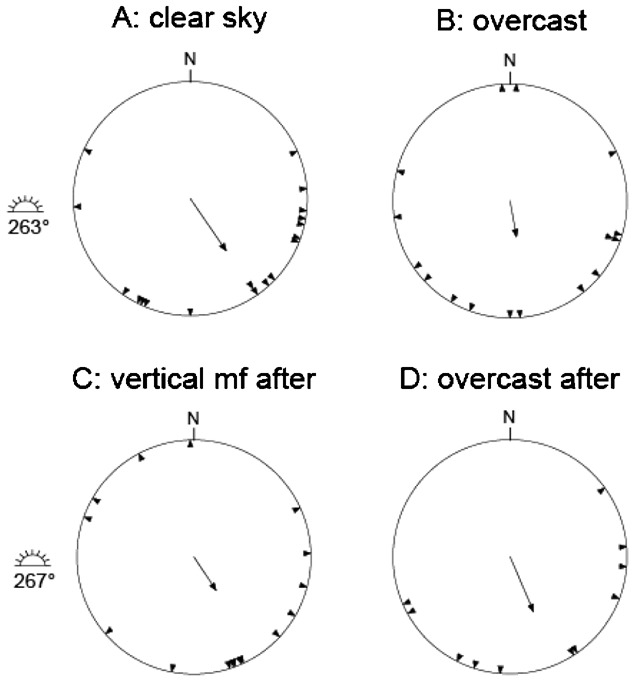
Results from our outdoor orientation cage experiments with juvenile sedge warblers, *Acrocephalus schoenobaenus* at Stensoffa Ecological Field Station in South Sweden during autumn migration. (A) Clear sky, (B) overcast sky, (C) exposure under clear skies without magnetic cues at vertical magnetic field vector, and (D) simulated overcast as a control after cue-conflict exposure. For further information, see legend for Fig. 1.

All three species showed preferred orientation towards southwest to southeast in experiments performed under natural clear skies and in the local geomagnetic field (Fig. 1A, Fig. 2A, Fig. 3A; Table 2). The mean orientation was significantly different from the position of the Sun in the middle of the test hour for sedge warblers (α: 145°, Sun: 264°; p<0.05, 95% CI ±33°; Fig. 3A; Table 2), dunnocks (α: 153°, Sun: 114°; p<0.05, 95% CI ±37°; Fig. 1A; Table 2), as well as for European robins (α: 250°, Sun: 268°; p<0.05, 95% CI ±13°; Fig. 2A; Table 2). The mean orientation under clear skies for all species was significantly different from the expected migratory directions as calculated for ringing recoveries (Fransson et al., 2008) of birds ringed in Sweden and recovered during the autumn migration and wintering areas (mean orientation based on ringing recoveries for sedge warbler: 193.9°; dunnock: 209.0°; European robin: 217.7°; p<0.05 in all cases, 95% CI). For the dunnock and the European robin, the preferred orientation recorded in the cages was directed between the expected migratory direction and the direction towards the sun during experiments (Table 2).

**Table 2.**
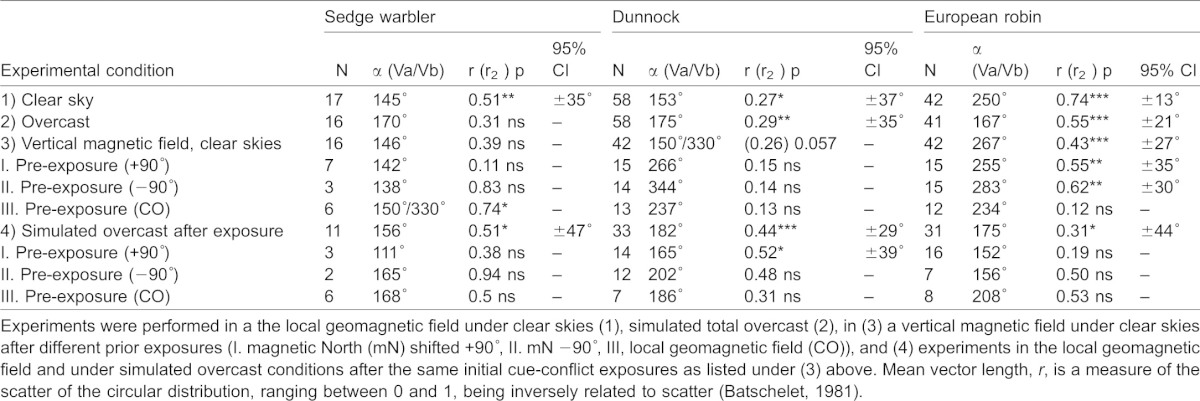
Number of birds, mean angle of orientation (α), vector length (*r*), significance level according to the Rayleigh test (p) ( **Batschelet, 1981**), and 95% confidence interval for orientation cage experiments with juvenile dunnocks, European robins and sedge warblers at Stensoffa Ecological Field Station in South Sweden during autumn migration

### Orientation under simulated overcast skies

The mean orientation recorded under overcast skies in the local geomagnetic field was for all three species directed south to southeast (Fig. 1B, Fig. 2B, Fig. 3B; Table 2). The mean orientation was not significantly different from the expected migratory direction for sedge warblers (α: 170°, ringing recoveries: 193.9°; p>0.05, 95% CI ±35°; Fig. 3B), and dunnocks (α: 175°, recoveries: 209.0°; p>0.05, 95% CI ±35°; Fig. 1B), but for European robins the mean orientation in cages under simulated overcast was directed more to the south than expected based on ringing recoveries (α: 167°, recoveries: 217.7°; p<0.05, 95% CI ±21°; Fig. 2B).

### Orientation after cue-conflict exposure

We found significant mean orientation (α = 267°, r = 0.43, p<0.001) under clear skies in a vertical magnetic field after cue-conflict exposure for the European robin (Fig. 2C; Table 2), with a higher scatter compared to the control tests. Furthermore, for the dunnock (Fig. 1C) and the sedge warbler (Fig. 3C) we found more scattered distributions resulting in mean orientations not significantly different from random (p>0.05, Table 2). When comparing the orientation recorded for European robins under clear skies in the local geomagnetic field (α = 250°) before the cue-conflict with orientation after under clear skies and in a vertical magnetic field (α = 267°) we found a significant difference between the two groups (U^2^ = 0.275, df = 42, p<0.01, Watson's U^2^-test) which was explained by a difference in scatter (t = 2.73, df = 82, p<0.05, Mardia's test for homogeneity of concentration parameters (Mardia, 1972)), suggesting no recalibration of compasses since an angular difference of 90° between the two groups were then to be expected.

According to Table 2, the mean orientation under simulated overcast skies and in the local geomagnetic field was directed south for the dunnock (α = 182°, Fig. 1D) and the European robin (α = 175°, Fig. 2D), and south south-east for the sedge warblers (α = 156°, Fig. 3D). For all species in these experiments there was no difference in the mean orientation under overcast as compared to the mean orientation in the overcast tests performed before the cue-conflict exposure (p>0.05 in all cases).

We have pooled the circular data for individuals independent of species for which we have successfully recorded the orientation under both clear skies in the local geomagnetic field and in a vertical magnetic field (incl +90°) after the cue-conflict exposure. In this experiment a re-calibration of the celestial compass would result in a +90° or −90° shift in mean orientation relative to the individually preferred orientation in the first experiment. For this analysis we pooled data for all individuals which were exposed to a magnetic field with the horizontal component shifted +90°, as compared to birds exposed to a magnetic field with mN shifted instead −90° at the cue-conflict, and no shift (exposure to clear sky and local geomagnetic field). The mean orientation in all of the three groups were not significantly different from random (mN+90°: α = 358°, r = 0.25, N = 35, p>0.05; mN−90°: α = 3°, r = 0.20, N = 31, p>0.05; local mN: α = 89°, r = 0.09, N = 29, p>0.05). Thus, the pooling of all data for each experimental category did not result in a clear picture revealing re-calibration of the magnetic or celestial compasses (a ±90° shift in orientation relative to previous test = 0°), but rather suggest that there was a tendency that there was no difference between the birds' orientation before and after the cue-conflict. However, the mean orientation when all tests were pooled were not significantly different from random (all: α = 10°, r = 0.16, N = 95, p = 0.082).

We analysed the possible re-calibration of the magnetic compass, by applying the same procedure as above. We pooled data from all individuals with successful registration of orientation under simulated overcast skies (before and after cue-conflict exposure). For each test the angular difference was calculated relative to the individually preferred orientation in the first experiment. A re-calibration of the magnetic compass based on geomagnetic information would then show by a ±90° shifted orientation in this comparison, independent of species preferred initial orientation. We found that the mean orientation of data from all our experimental birds which prior to the cue-conflict experiment experienced a +90° shift of the horizontal component of the magnetic field, were not significantly different from random (α = 75°, r = 0.21, N = 27, p>0.05). This was true also for the group experiencing a −90° shift of the magnetic field (α = 348°, r = 0.33, N = 19, p>0.05). The group of birds that did not experience any cue-conflict, but were given access to the surrounding landscape and the local geomagnetic field during the pre-exposure, were also not different from random (α = 274°, r = 0.33, N = 24, p>0.05). Unfortunately the sample sizes were still limited for these groups, as we could only use birds with significant mean orientation in both tests. Furthermore, we did not find a significant mean orientation if all three groups were pooled (α = 336°, r = 0.12, N = 70, p>0.05). Thus, our pooled analyses could not demonstrate any re-calibration of the celestial or magnetic compass in our experimental birds, but did not confirm a persistent celestial or magnetic compass orientation between tests either. The reason for this lack of persistence in magnetic compass orientation may be a larger scatter in orientation when visual information was not available, small sample size and/or a high variation in individually preferred orientation between tests.

### Effect of skylight polarization on orientation

**Fig. 4.**
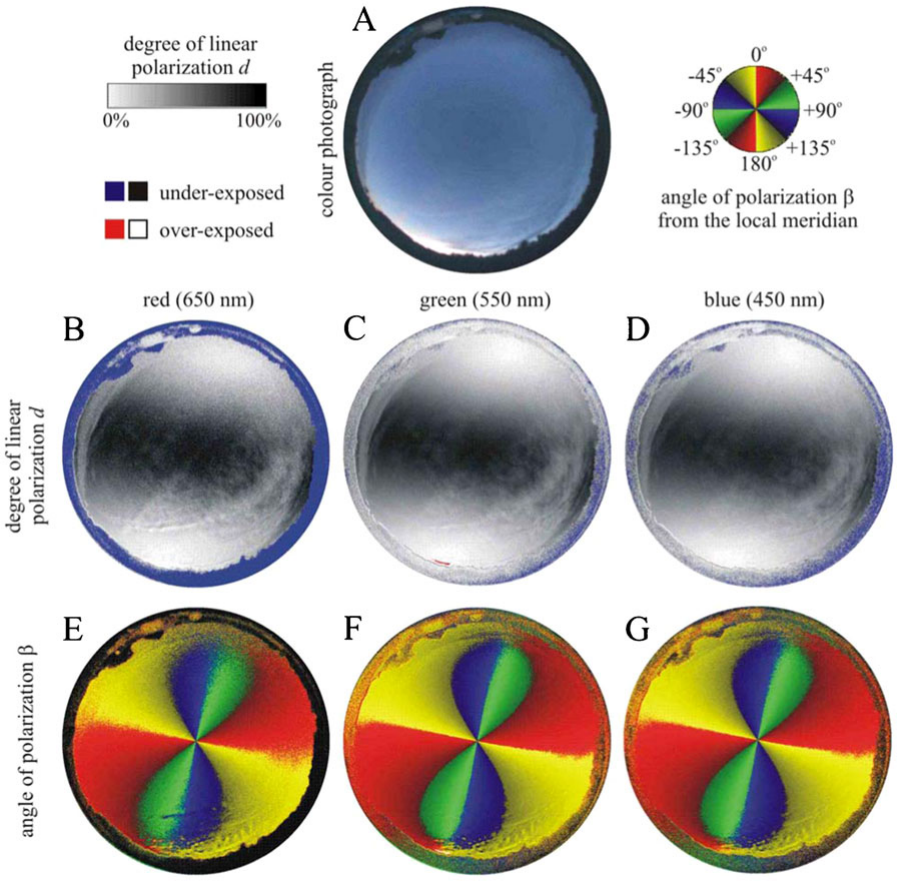
Photograph and patterns of the degree of linear polarization *d* and the angle of polarization β (clockwise from the local meridian) of the cirrus-clouded sky no. 345 measured by full-sky imaging polarimetry (Table 3). (A) Colour photograph. (B–D) Patterns of the degree of linear polarization *d*. (E–G) Patterns of the angle of polarization β of the natural sky as measured by full-sky imaging polarimetry in the red (650 nm), green (550 nm) and blue (450 nm) parts of the spectrum.

**Fig. 5.**
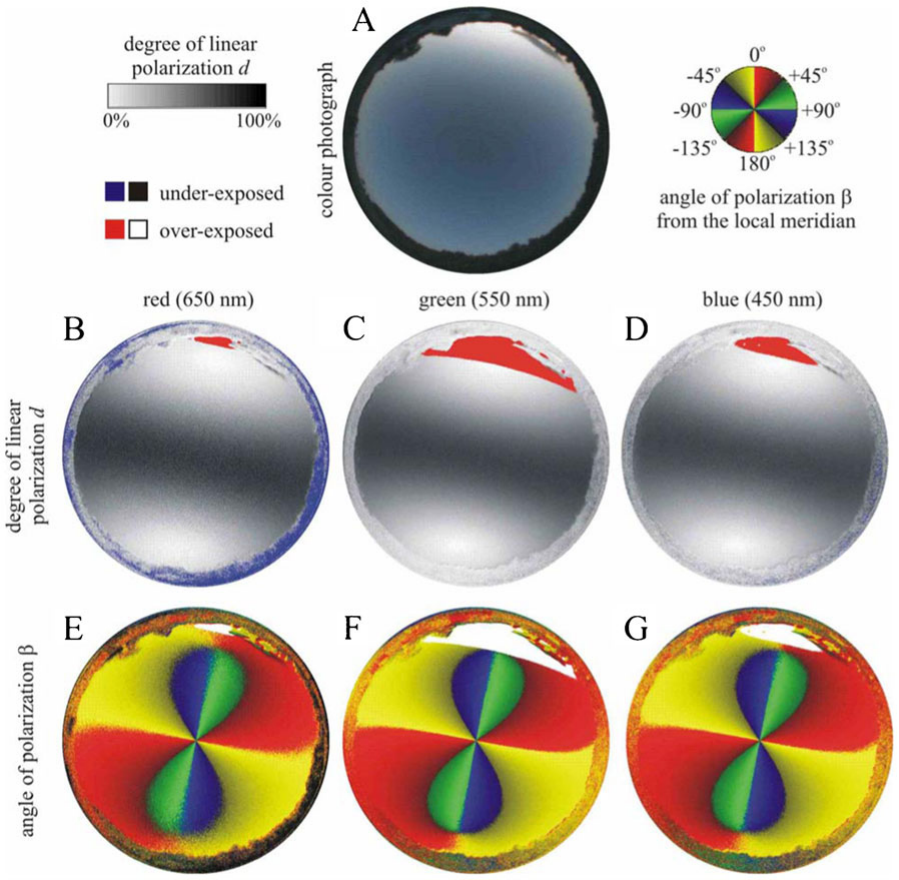
As Fig. 4 for the clear sky no. 402

**Fig. 6.**
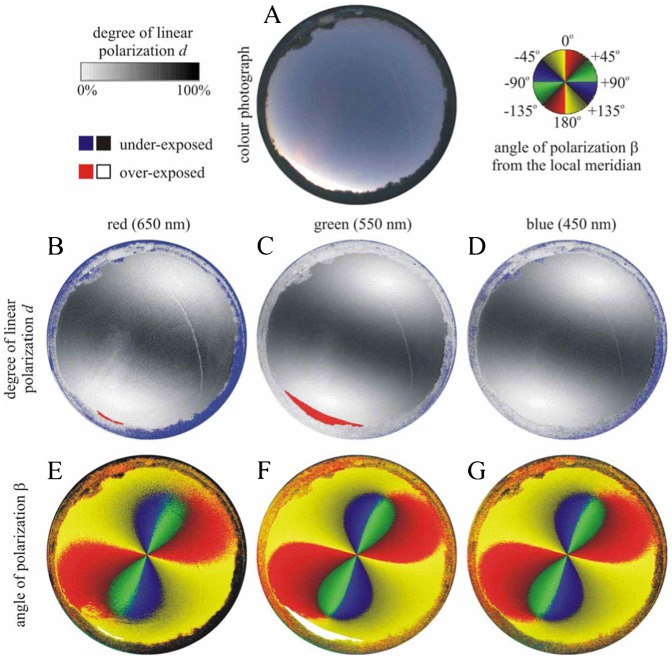
As Fig. 4 for the nearly clear sky no. 618

**Fig. 7.**
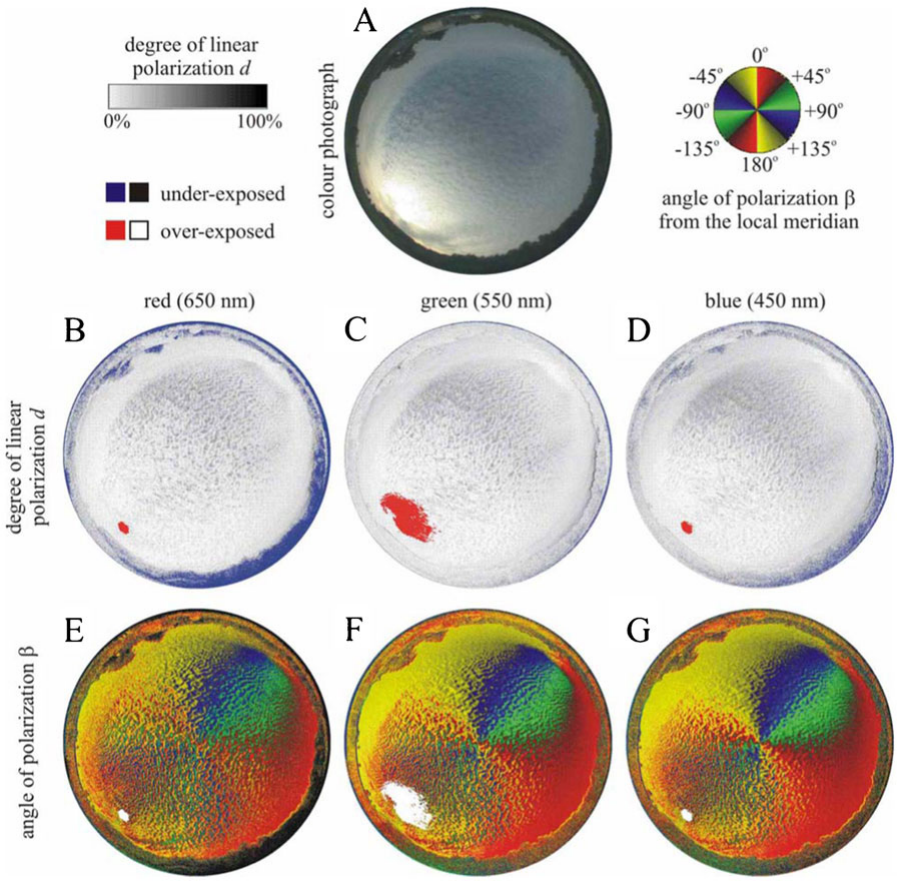
As Fig. 4 for the strongly cloudy sky no. 324

**Fig. 8.**
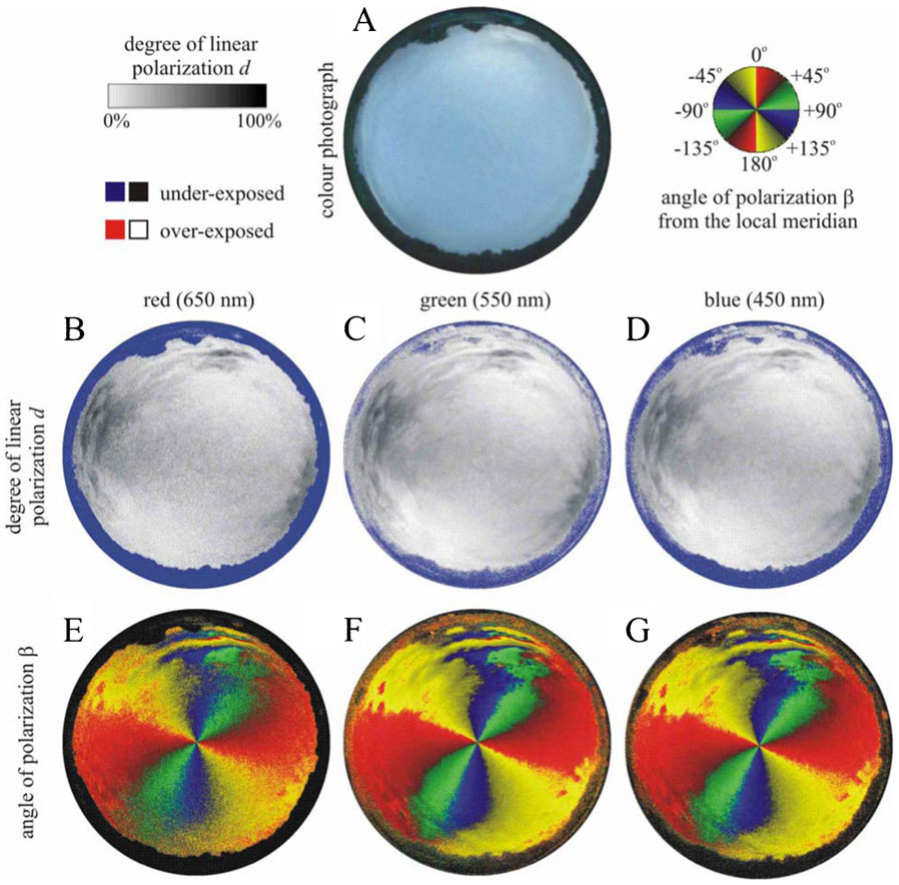
As Fig. 4 for the totally cloudy (overcast) sky no. 357

**Fig. 9.**
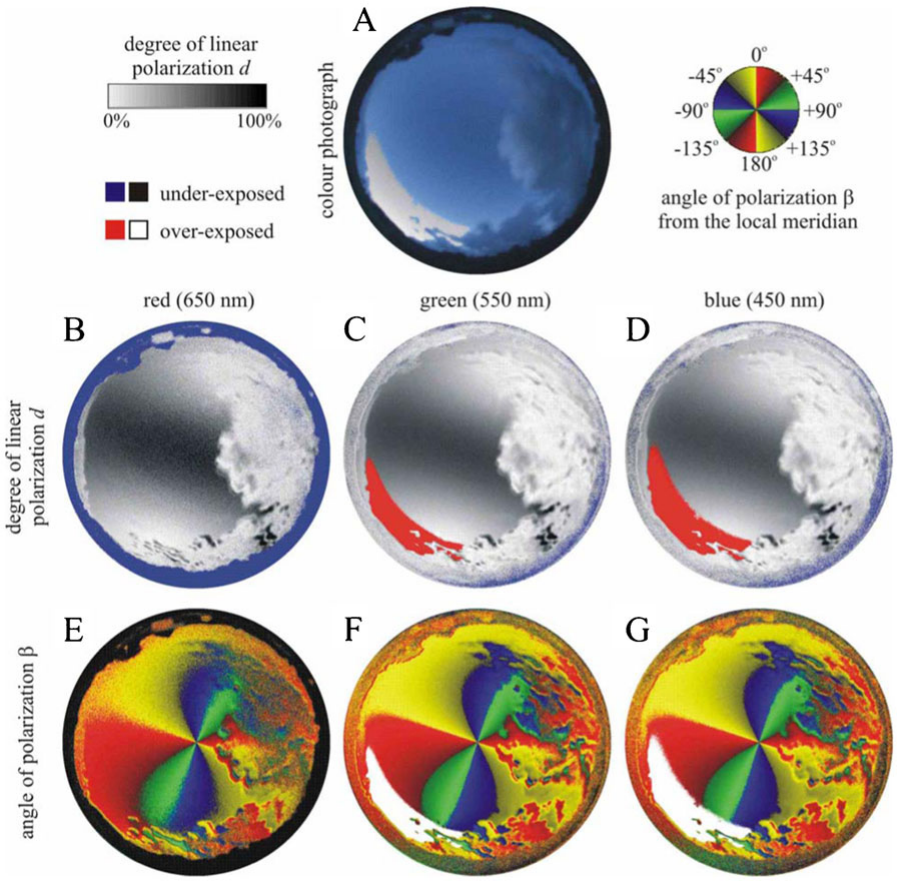
As Fig. 4 for the partially cloudy sky no. 454

**Fig. 10.**
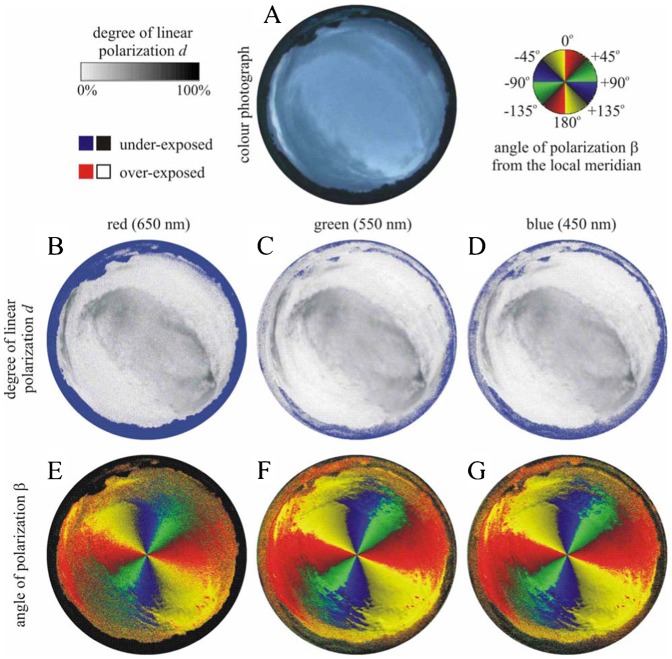
As Fig. 4 for the totally cloudy sky no. 490 with two cloud layers (one layer centrally and another one peripherally)

**Fig. 11.**
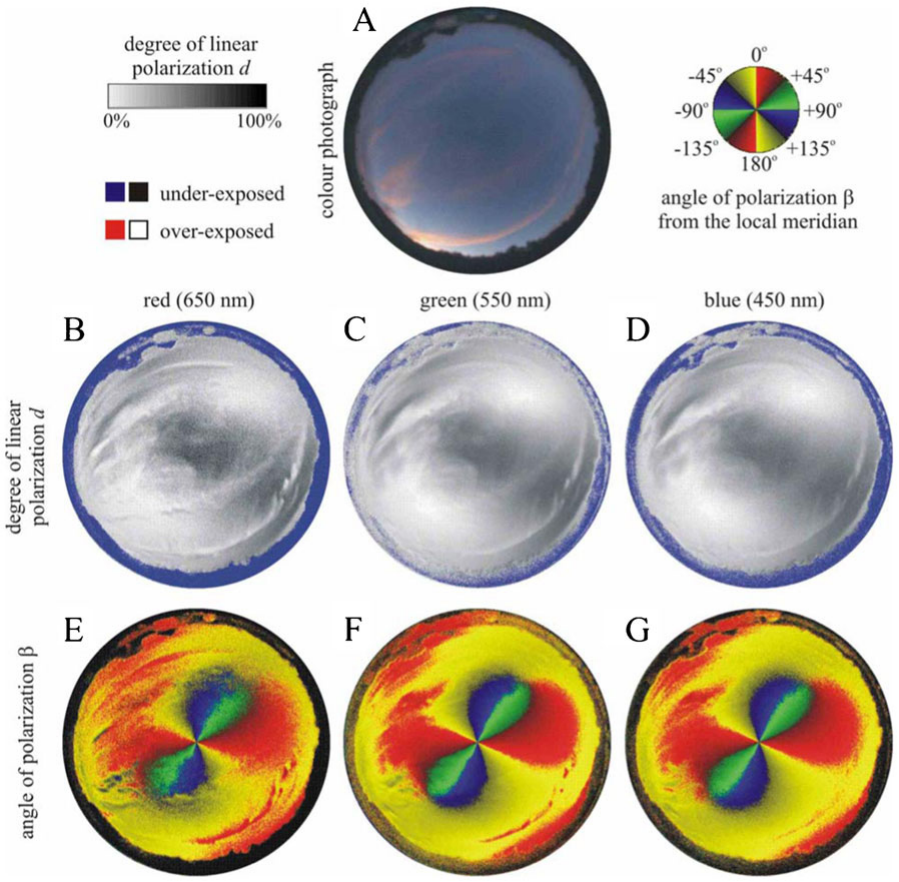
As Fig. 4 for the sky no. 584 with cirrus clouds

We measured the availability of polarized light from the sky as a compass cue in our experiments. Table 3 shows the average ± standard deviation of the degree of linear polarization *d* and its maximum *d*_max_ measured by full-sky imaging polarimetry in the red (650 nm), green (550 nm) and blue (450 nm) spectral ranges during the bird orientation experiments. The average of *d* was calculated for the full sky, while *d*_max_ was detected along a celestial great circle at 90° from the Sun. As examples for the sky conditions during the bird orientation experiment, Figs 4–11 show the photograph and the patterns of the degree of linear polarization *d* and the angle of polarization β of clear (Figs 4–6) and more or less cloudy (Figs 7–11) skies measured by full-sky imaging polarimetry. The general features of sky polarization were the following: (i) The more cloudy was the sky, the lower was the degree of skylight polarization. (ii) The celestial pattern of the angle (direction) of polarization was practically independent of the cloudiness. Consequently, if a bird was able to perceive the strong or weak skylight polarization, it could orient on the basis of the celestial pattern of the direction of polarization.

**Table 3.**
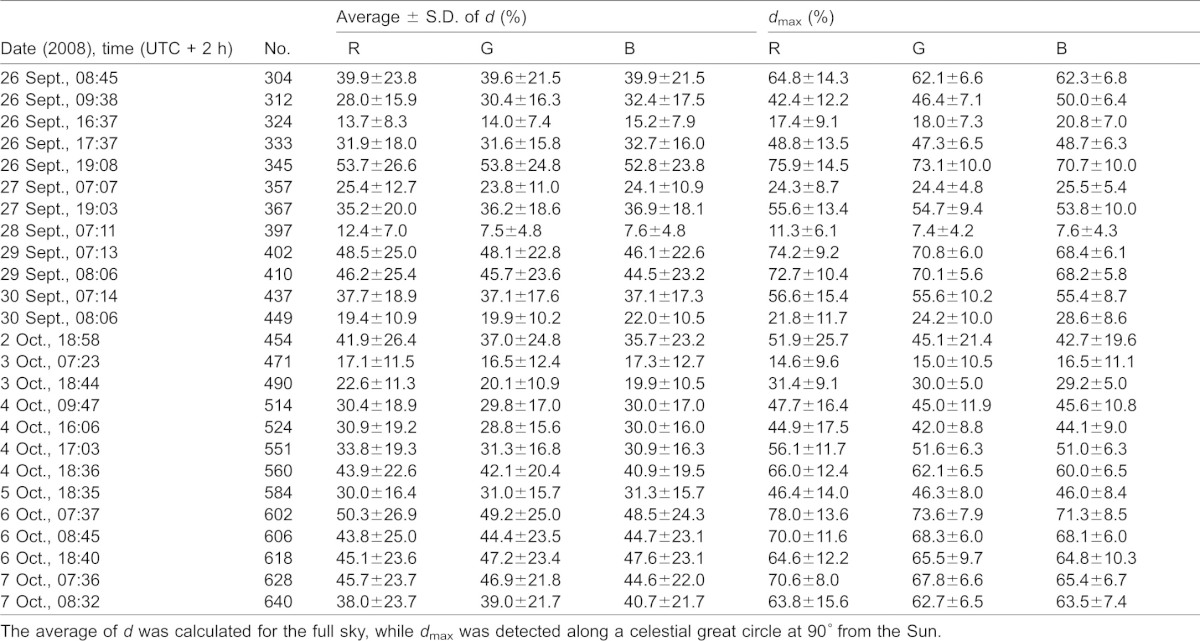
Date (2008) and time (UTC + 2 h), sky number (No.), average ± standard deviation (S.D.) and *d*_max_ of the degree of linear polarization *d* (%) measured by full-sky imaging polarimetry in the red (R, 650 nm), green (G, 550 nm) and blue (B, 450 nm) parts of the spectrum during our bird orientation experiment

**Fig. 12.**
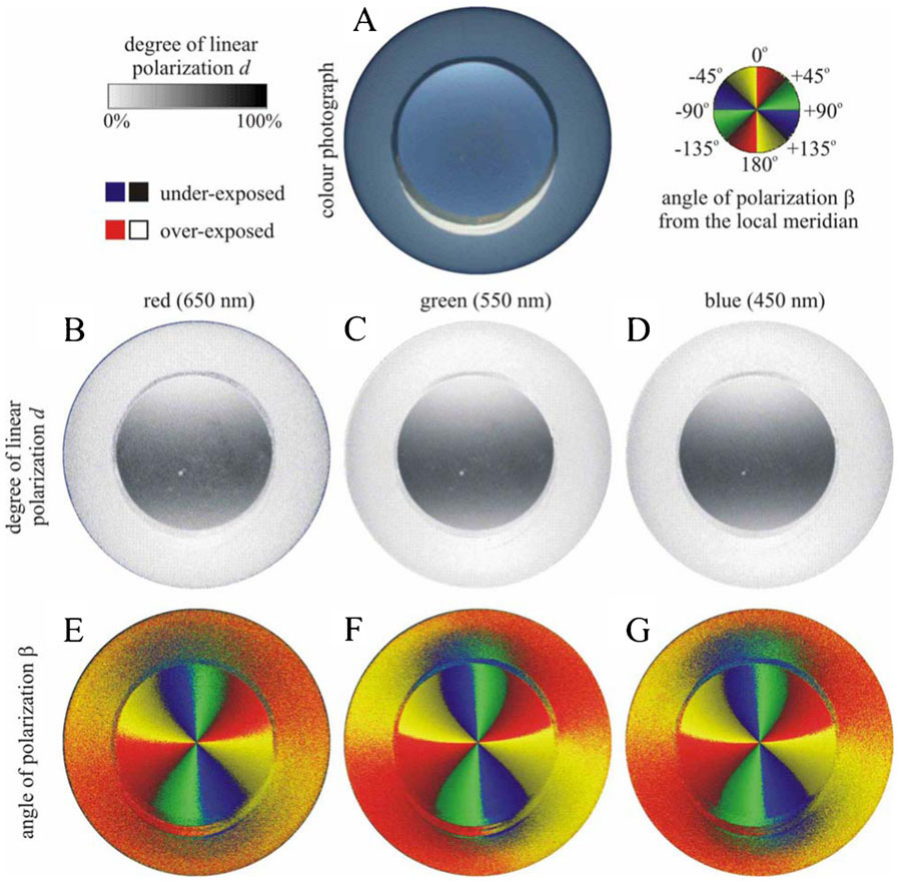
As Fig. 4 when the polarimeter was looking at the clear sky, out of the Emlen funnel used in our bird orientation experiment

As an example, Fig. 12 shows the distribution of celestial polarization measured with a polarimeter that was looking out from the Emlen funnel used in the bird orientation experiment. According to our measurements, the degrees of polarization of light reflected from the matte white bird scratch registration paper (Tipp-Ex or Thermo paper) covering the inside of the funnel were very low (*d*<5%). On the other hand, the pattern of the direction of polarization of this registration paper was practically the same as that of the actual sky (in other words, the former pattern was the continuation of the latter). Hence, this very weak polarization pattern of the registration paper could not disturb the orientation of birds tested: this pattern (i) was either not sensed by birds due to its very low *d*-values, or (ii) if sensed, it could be interpreted by birds as the continuation of the celestial pattern of direction (angle β) of polarization.

**Fig. 13.**
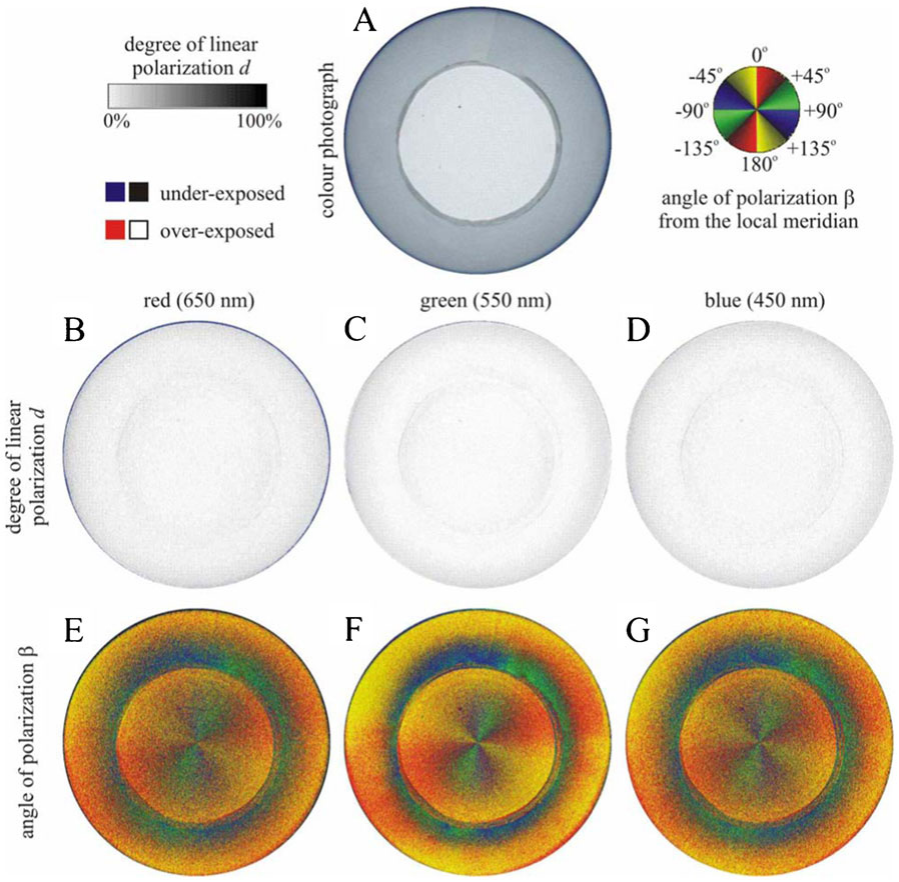
As Fig. 12 when the Emlen funnel was covered by a 2 mm thick depolarizing milky plastic board

Fig. 13 shows the polarization patterns seen from the Emlen funnel when it was covered by a 2 mm thick depolarizing milky plastic board. The degree of polarization was very low (*d*<5%) in the whole upper hemisphere due to the depolarizer. The pattern of the angle of polarization β was similar to that of the clear sky. Hence, if a bird could sense this very weak polarization, it could orient on the basis of the β-pattern, since from this pattern (the mirror symmetry axis of which is parallel to the solar–antisolar meridian) the direction of the solar–antisolar meridian can be determined.

The polarization measurements collected during the experiments showed clearly the different access to information from the pattern of skylight polarization to the birds in our experiments. Despite both magnetic and polarization information being available, the birds did not recalibrate their magnetic or celestial compasses.

## DISCUSSION

In our cage experiments designed to study in a comparative way the calibration of celestial and magnetic compasses in songbirds with different migration strategies and distances of migration we did not find any support for re-calibration of the magnetic or celestial compass. All three species of songbirds (dunnock, European robin and sedge warbler) reacted in a similar way to the cue-conflict situations by not shifting their orientation relative to celestial or magnetic cues available. Hence, we could not confirm the findings by Muheim et al. (Muheim et al., 2006a; Muheim et al., 2007; Muheim et al., 2009), where two species of North American sparrows have been demonstrated to recalibrate their magnetic compass on the basis of polarized light from the sky near the horizon.

To avoid the risk of introducing optical artefacts by our experimental set-up, we measured the availability of linearly polarized light inside our experimental cages and at the site where our cages were located during the experiments under different cloud conditions. We found that linearly polarized skylight with high enough degrees of polarization was readily available in the clear sky tests from our study location. The Tipp-Ex papers used in the experiments reflected weakly polarized light (d<5%), but the pattern of angle of polarization extended from the freely seen sky to the covered part of the field of view (Fig. 13), so the disturbance from this reflection could be assumed minimal. Hence, we can confidently state that the access of polarized skylight would have enabled the birds to recalibrate their magnetic compass, in case they would have been inclined to do so. On the other hand, although the diffusers (2 mm milky Plexiglas) transmitted the celestial pattern of the angle of polarization similar to that of the clear sky, the degree of polarization was extremely low (<5%). Such a weak polarization is not perceivable by any known polarization-sensitive animal (Horváth and Varjú, 2004). Even the field cricket (*Gryllus campestris*), the most polarization-sensitive known species, is not able to sense such low degrees of polarization (Herzmann and Labhart, 1989; Labhart, 1996). Thus, the depolarizing milky plastic board used in our experiment should completely abolished polarization information for our test birds.

Our results have added data on three species of songbirds, studied in Scandinavia, which have not recalibrated their magnetic compass on the basis of linearly polarized skylight during migration. This means that currently the great majority of cue-conflict experiments have not lead to recalibrations (Wiltschko et al., 2008; Gaggini et al., 2010; Chernetsov et al., 2011; Schmaljohann et al., 2013; this study), while it is so far only two species of sparrows studied in North America, which have shown this effect (Muheim et al., 2006a; Muheim et al., 2007; Muheim et al., 2009). Our study was performed in a similar experimental situation, including the same type of registration cages as in Muheim et al. (Muheim et al., 2006a), and still there has not been any confirmed re-calibration in our birds. The experiments which have led to recalibration of the magnetic compass have both been performed in North America with North American songbird species (Muheim et al., 2006a; Muheim et al., 2009), while the rest of the experiments have been performed at three different locations in Europe (Russia, Rybatchy (Chernetsov et al., 2011), Italy (Gaggini et al., 2010), Germany (Schmaljohann et al., 2013), Sweden (this study)) and in one location in Australia (Wiltschko et al., 2008). It has previously been argued that the crucial celestial information necessary for re-calibration of the magnetic compass is the pattern of polarized skylight near the horizon (Muheim et al., 2006a; Muheim et al., 2006b; Muheim et al., 2009). In our experiments we could demonstrate that the birds had access to polarized skylight during cue-conflict exposure (visible from inside the cue-conflict exposure cages) as well as during the cage experiments, and that the simulated overcast situation omitted this celestial information from the birds' view. Despite this we could not find a clear change in orientation before and after the cue-conflict exposure. Thus, we must conclude that the birds, and especially the European robin, for which we had a sufficiently large sample size, did not show any evidence for re-calibration, and the potential influence of optical artefacts (Horváth and Varjú, 2004; Muheim, 2011) were kept to a minimum.

It is likely that the different responses of the currently available cue-conflict experiments performed with caged songbird migrants, and in release experiments have a biological and ecological explanation. In our case the visibility of the polarized skylight near the horizon was controlled for, and is likely not involved in the outcome of our experiments. It has been argued that also in Australia the skylight near the horizon was available to the experimental birds (Wiltschko et al., 2008), while it may not have been in previous cage experiments and possibly may have influenced the outcome of these (Muheim et al., 2006b).

The most obvious difference between studies with and without re-calibration responses is the location of the experiments (North America vs. Europe, Australia). One may wonder if possibly the geographic area may influence how birds evolved to migrate within a certain geographical range of the globe, may adapt their compass use and integration between compasses based on the availability and reliability of the compass information in different areas. In North America migratory birds are to a large extent exposed to large variations in declination, i.e. angular difference between geographic and geomagnetic North, across nearby areas, especially in the high Arctic, where declination may change fast when crossing longitudes on migration flights (Åkesson et al., 2005). The songbirds migrating across the North American continent may therefore have adapted to a system of re-calibration of compass information to cope with these declination variations met during migrations (Able and Able, 1990a; Able and Able, 1993; Able and Able, 1995). They may further use information from the declination to define their longitude on migrations (Åkesson et al., 2005; Gould, 1998).

Despite the fact that we could not confirm re-calibration of the magnetic compass in our experimental birds, the current discrepancies between cage experiments performed with different species of songbirds in different geographical areas call for further attention. We believe that answers related to geographical area, avian migration ecology and potentially also experimental design should be sought for. We therefore welcome systematic and careful experimentation performed under strictly controlled conditions to investigate the cue-conflict effects in songbird orientation, and to especially study the possible effect of geographical location of study site on re-calibration.

## MATERIALS AND METHODS

### Study species

Three groups of young dunnocks (n = 48), European robins (44) and sedge warblers (24) identified to species and age by plumage according to Svensson (Svensson, 1992). The experimental birds were captured with mistnets at inland stopover sites near (<2 km) Stensoffa Ecological Station (55°42′N, 13°25′E) in South Sweden during autumn migration in September and October 2008. At the same sites in October 2009 a further 17 dunnocks were caught and tested in control experiments under natural clear and simulated overcast skies. We performed orientation cage experiments involving a series of four tests for each individual bird performed either at local sunrise (dunnock) or sunset (European robin and sedge warbler) (for method, see Åkesson, 1994) to study their compass orientation and after-effects on the birds preferred orientation after a cue-conflict exposure.

We recorded the birds' body mass to the nearest 0.1 g with a Pesola spring-balance (60 g) and classified the fat levels according to a 10-graded visual scale for fat classification (Pettersson and Hasselquist, 1985) (extended with 3 grades at Falsterbo Bird Observatory) at capture and immediately after the cage experiments. The visually classified fat levels were used to identify if the birds were prepared for migration. Only birds with fat scores >2 at capture were selected for experiments. All birds were released at the site of experiments located <2 km of capture site after the experimental series were finished.

Dunnocks are predominantly diurnal migrants and birds from Scandinavia migrate towards southwest to wintering areas in Southern France and Northern Spain (Roos, 1984; Fransson et al., 2008). Both European robins and sedge warblers are nocturnal migrants (Cramp, 1988; Cramp, 1992). The European robins captured in Sweden are wintering in south-western Europe, while sedge warblers migrate to tropical West Africa (Moreau, 1972; Zink, 1973). For each species the expected autumn migration course for birds ringed in Sweden as calculated along an initial great circle route were directed towards geographic south-west to south [dunnocks: 209.0°, r = 0.96, n = 56, European robins (juv): 217.7°, r = 0.93, n = 358, sedge warblers (juv): 193.9°, r = 0.95, n = 348 (Fransson et al., 2008)].

### Experimental procedure

The migratory orientation of individual birds was recorded in circular cages, so-called Emlen funnels (Emlen and Emlen, 1966) (lined with Tipp-Ex paper or Thermo paper), allowing the birds to see with an approximately 140° field of view onto the sky around zenith. The experiments were performed outdoors during the autumn migration period at Stensoffa Ecological Station in South Sweden (for methods, see Åkesson, 1994). The mean angle of orientation of individual birds was recorded for one hour in each test condition. To simulate overcast conditions, the top of the cage was covered with a 2 mm thick diffusing milky Plexiglas sheet during overcast experiments, permitting no celestial information to enter the cage. The mean orientation of each bird was recorded under the following experimental conditions: (1) natural clear skies (cloud cover 0–1/8) in the local geomagnetic field, and (2) simulated overcast skies under diffusing Plexiglas in the local geomagnetic field.

In cue-conflict exposures, the birds were exposed to a cue-conflict during one hour (starting ca. 90 min before local sunset) at sunset as well as during the following sunrise (exposure started at sunrise) in cages from where they had access to a full view of the landscape and sky around them and thereafter their orientation was recorded in circular cages for which experiments were initiated after ca 30 min under (3) simulated overcast skies in the local geomagnetic field, and (4) natural clear skies in a vertical magnetic field. The relative order of tests 1 and 2, as well as 3 and 4 was randomly selected for the individual birds.

### Evaluation of orientation data and statistics

Based on the bird's activity in the cage as recorded by claw marks in the white pigment of the Tipp-Ex and Thermo papers, we visually estimated the mean angle of orientation relative to geographic north using a classification method described by Mouritsen (Mouritsen, 1998) and first developed by Rabøl (Rabøl, 1979). We modified the method by using the limit for a minimum of 40 registrations similar to what we have been using in previous experiments (Åkesson, 1994; Åkesson et al., 2001), enabling us to compare the activity levels among tests. The direction associated with the highest concentration of scratch marks was visually inspected and marked for each registration paper on a light table. By using a 360° protractor in the funnel together with the paper, the position of the chosen direction was translated to the closest 5°. The scratch distribution was then evaluated with concentration and activity indices on a 0–4 scale (Mouritsen, 1998). The activity index was set by estimating the amount of scratches on the whole paper made during one hour, where 0 is <40 scratches and 4 is >2000 scratches. The concentration index indicates the uncertainty, or scatter, of the direction and was estimated within how many degrees 10 people would point out the bird's direction (see Table II in Mouritsen (Mouritsen, 1998)). Experiments were included in further statistical tests when both indices were ≧1, and the sum of them was ≧3. Number of experiments classified as active, inactive, disoriented and included in the analyses, are given in Table 1. For individuals with a significant axial mean orientation (56 out of 483 experiments), we used only the side of the axis with the majority of the registrations for further statistical analyses. We used circular statistics to calculate the mean orientation for each species and experimental condition, and the Rayleigh test to analyse if the mean orientation differed from a random distribution (Batschelet, 1981). Differences between groups were compared with Watson's U^2^-test (Batschelet, 1981) and Mardia's test for homogeneity of concentration parameters (Mardia, 1972). We used 95% Confidence Interval (95% CI (Batschelet, 1981)) to analyse if the mean orientation differed from the expected migratory directions along an initial great circle route for birds ringed in Sweden (Fransson et al., 2008) or from the Sun position during the experiments.

### The Earth's magnetic field and Sun's azimuth

The geomagnetic parameters, i.e. total field intensity, inclination and declination, were calculated by GEOMAGIX based on the model for the World Magnetic Model (WMM 2005) for the test site at 1 September 2008. The azimuth position of the Sun in the middle of the test hour was calculated relative to geographic north for each date and time of experiments. The mean Sun azimuth angle was calculated for each experimental group based on the Sun position calculated for individual tests.

### Measurements of skylight polarization

The skylight polarization was measured by full-sky imaging polarimetry. This technique has been described in detail by Gál et al. and Horváth and Varjú (Gál et al., 2001; Horváth and Varjú, 2004). A 180° field of view (full sky) was ensured by a fish-eye lens (Nikon-Nikkor with *F* = 2.8, focal length  =  8 mm). This lens had a built-in rotating wheel mounted with three broadband (275–750 nm) neutral-density linearly polarizing filters (Polaroid HNP'B) with three different polarization axes (90°, 135° and 0° from the radius of the wheel). The detectors were the red-, green- and blue-sensitive CMOS pixels of a Nikon D3 digital camera. For a given sky, three polarization photographs (12 megapixel raw images in .NEF format) were taken at the three different orientations of the transmission axis of the linear polarizers. The camera was set up on a tripod such that the optical axis of the fish-eye lens was vertical, pointing to the zenith. After computer evaluation of these three polarization pictures of a given sky, the patterns of the intensity *I*, degree of linear polarization *d*, and angle of polarization β (measured clockwise from the local meridian) of skylight were determined as colour-coded, two-dimensional, circular maps, in which the center is the zenith, the perimeter is the horizon, and the zenith angle θ is proportional to the radius from the zenith (zenith: θ = 0°, horizon: θ = 90°). These patterns were obtained in the red, green, and blue spectral ranges, in which the CMOS pixels of the camera had maximal sensitivity. The degree *d* and the angle β of linear polarization were measured by our polarimeter with an accuracy of Δ*d* = ±2% and Δβ = ±2°, respectively. The degree of polarization *d* of skylight was averaged (average ± standard deviation) either for the whole sky, or for a narrow celestial band of 5° angular width at an azimuthal distance of 90° from the Sun (when the maximum of *d* was determined).

With our full-sky imaging polarimeter we also measured the reflection–polarization characteristics of the inside of the Emlen funnel used in the bird orientation experiments. In this case the funnel was positioned over the polarimeter that the fisheye lens was placed at the site of the head of the experimental bird. Thus, the polarimeter recorded what a bird can see when looking at the sky from the test cage (funnel).
